# Infant and child mortality in relation to malaria transmission in KEMRI/CDC HDSS, Western Kenya: validation of verbal autopsy

**DOI:** 10.1186/s12936-018-2184-x

**Published:** 2018-01-18

**Authors:** Nyaguara O. Amek, Annemieke Van Eijk, Kim A. Lindblade, Mary Hamel, Nabie Bayoh, John Gimnig, Kayla F. Laserson, Laurence Slutsker, Thomas Smith, Penelope Vounatsou

**Affiliations:** 1 Kenya Medical Research Institute, Centre for Global Health Research, P.O. Box 1578, Kisumu, Kenya; 2 Swiss Tropical and Public Health Institute, Socinstr. 57, P.O. Box, 4002 Basel, Switzerland; 3 University of Basel, Petersplatz 1, P.O. Box, 4003 Basel, Switzerland; 4 Centers for Disease Control and Prevention, 1600 Clifton Rd, Atlanta, GA 30301 USA

**Keywords:** Childhood mortality, Bayesian inference, Malaria entomology data, Verbal autopsy, Health and demographic surveillance system

## Abstract

**Background:**

Malaria transmission reduction is a goal of many malaria control programmes. Little is known of how much mortality can be reduced by specific reductions in transmission. Verbal autopsy (VA) is widely used for estimating malaria specific mortality rates, but does not reliably distinguish malaria from other febrile illnesses. Overall malaria attributable mortality includes both direct and indirect deaths. It is unclear what proportion of the deaths averted by reducing malaria transmission are classified as malaria in VA.

**Methods:**

Both all-cause, and cause-specific mortality reported by VA for children under 5 years of age, were assembled from the KEMRI/CDC health and demographic surveillance system in Siaya county, rural Western Kenya for the years 2002–2004. These were linked to household-specific estimates of the *Plasmodium falciparum* entomological inoculation rate (EIR) based on high resolution spatio-temporal geostatistical modelling of entomological data. All-cause and malaria specific mortality (by VA), were analysed in relation to EIR, insecticide-treated net use (ITN), socioeconomic status (SES) and parameters describing space–time correlation. Time at risk for each child was analysed using Bayesian geostatistical Cox proportional hazard models, with time-dependent covariates. The outputs were used to estimate the diagnostic performance of VA in measuring mortality that can be attributed to malaria exposure.

**Results:**

The overall under-five mortality rate was 80 per 1000 person-years during the study period. Eighty-one percent of the total deaths were assigned causes of death by VA, with malaria assigned as the main cause of death except in the neonatal period. Although no trend was observed in malaria-specific mortality assessed by VA, ITN use was associated with reduced all-cause mortality in infants (hazard ratio 0.15, 95% CI 0.02, 0.63) and the EIR was strongly associated with both all-cause and malaria-specific mortality. 48.2% of the deaths could be attributed to malaria by analysing the exposure–response relationship, though only 20.5% of VAs assigned malaria as the cause and the sensitivity of VAs was estimated to be only 26%. Although VAs assigned some deaths to malaria even in areas where there was estimated to be no exposure, the specificity of the VAs was estimated to be 85%.

**Conclusion:**

Interventions that reduce *P. falciparum* transmission intensity will not only significantly reduce malaria-diagnosed mortality, but also mortality assigned to other causes in under-5 year old children in endemic areas. In this setting, the VA tool based on clinician review substantially underestimates the number of deaths that could be averted by reducing malaria exposure in childhood, but has a reasonably high specificity. This suggests that malaria transmission-reducing interventions such as ITNs can potentially reduce overall child mortality by as much as twice the total direct malaria burden estimated from VAs.

**Electronic supplementary material:**

The online version of this article (10.1186/s12936-018-2184-x) contains supplementary material, which is available to authorized users.

## Background

Under-five mortality still remains a major public health problem in sub-Saharan Africa (SSA). Of the 8.8 million global annual under-five deaths, about 50% occur in SSA. In Kenya, one in twelve children (84 per 1000 live births) dies before their fifth birthday [1]. On a global scale, most under-five (childhood) deaths have been attributed to pneumonia, diarrhoea, malaria, neonatal sepsis, malnutrition, preterm delivery and asphyxia at birth [2]. Most of these conditions/diseases are either preventable or treatable with minimum interventions [3]. Scaling up of malaria interventions, including use of insecticide-treated nets (ITNs), artemisinin-based combination therapy (ACT) and intermittent preventive treatment (IPT) both in pregnancy and infancy, probably accounts for much of the recent dramatic declines in the mortality and hospital admissions in African children [4–8]. Malaria/or malaria associated conditions are still thought to be one of the leading causes of pediatric morbidity and mortality [9], but there is controversy about the overall size of the remaining burden [10–12].

Due to poor vital registration systems [13] and the fact that most children die at home without any contact with the health system, estimates of cause-specific mortality rates in SSA are mainly inferred using verbal autopsy data (VA) [14–18]. However, there is no gold standard to validate malaria deaths in VA. Some studies have compared hospital-based causes of deaths with the ones assigned by VA but have shown poor performance [19–22]. In the coastal region of Kenya a study comparing hospital deaths-based causes of death in children with the ones assigned by VA found the sensitivity of VA in identifying malaria deaths to be less than 50% [19]. At the same time, in malaria endemic areas, over reporting of malaria deaths is common because it shares symptoms with other diseases such acute respiratory infection including pneumonia or meningitis which are often assigned as malaria using VA [19]. In particular febrile illness with no other confirmed aetiology is usually recorded as malaria in VA [14].

An alternative approach to estimate the malaria- attributable burden is to base this on the relationship between all-cause mortality rates and malaria exposure or transmission. Exposure is ideally measured via the entomological inoculation rate (EIR), but because accurate estimates of both the EIR and mortality rates require very large amounts of data such analyses are generally based on between-site ecological analyses of convenience samples from a small number of sites, mostly Health and Demographic Surveillance Systems (HDSS) [22–25]. One such analysis found all-cause child mortality rates across Africa to be significantly associated with EIR in infants but no clear trend was observed in children (12–59 months) [24].

The malaria exposure-mortality relationship has also been analysed using mortality data from Demographic and Health Surveys (DHS) from Mali [26]. DHS are national surveys carried out in a standardized way at specific time periods and provide child mortality data from much wider areas than HDSS sites and can be adjusted for climatic and environmental factors. However, the Mali analysis found no clear relationship between malaria transmission and mortality with data on malaria prevalence in humans from the Mapping Malaria Risk in Africa (MARA) database. This could have been because the two datasets are spatially and temporally mis-aligned and contain data from different age groups of hosts. Similarly, since malaria transmission varies considerably over small areas, there may be less variation in exposure between different regions of a country than within one small area. Where this is the case, spatial averaging either of the exposure or the response biases estimates of exposure–response relationships towards zero.

An approach that minimizes such averaging effects is to model household-level entomological exposures across single HDSS sites, using Bayesian hierarchical modelling techniques to estimate the exposure–response relationships in a way that allows for the considerable uncertainty in such exposure estimates. The Malaria Transmission Intensity and Mortality Burden across Africa (MTIMBA) project is carrying out such analyses of data from a number of sites across Africa. One completed analysis within this project, of longitudinal data from Rufiji HDSS, found no association between all-cause mortality in under-5 year old children and malaria transmission intensity once ITN use was taken into account [27]. The present study, also under the overall MTIMBA umbrella, is an analysis of all-cause and malaria specific child mortality in relation to estimated EIR from KEMRI/CDC HDSS site in Western Kenya. The analysis is extended to provide estimates of malaria-attributable mortality with those derived from clinician-coded VAs. This represents a novel approach for validating the diagnosis of malaria in VAs, and for estimating the overall burden, both of direct malaria mortality, and of all-cause mortality attributable to malaria.

## Methods

### Study area and population

The KEMRI/CDC health and demographic surveillance system (HDSS) is located in three regions namely Asembo (Rarieda Division, Bondo District), Gem (Yala and Wagai Divisions, Siaya District) and Karemo (Karemo, Division, Siaya District) in Siaya county, rural Western Kenya. During the study period, the HDSS operated in Asembo and Gem, an area of approximately 500 km^2^ with a population of 135,000 living in 33,990 households in 21,477 compounds in 217 villages. The residents of the study area are predominantly from the Luo ethnic group, and derive their livelihood mainly from subsistence farming. This area is one of the most deprived in Kenya with over 66% of the inhabitants living below the poverty level [28]. The study area has high (243 per 1000 live births) under-five mortality [29] and malaria infection is mainly transmitted by *Anopheles gambiae* s.l. [30]. An insecticide-treated mosquito nets (ITN) trial conducted from 1996 to 2002 in the area reduced malaria transmission by 90% [31, 32]. However, despite the continued high prevalence of ITN use and a relatively low EIR of about seven infectious bites per year [33], malaria prevalence is still high and is thought to be the main cause of child mortality [29].

### Mortality data

The HDSS routinely conduct household surveillance through house-to-house interviews by trained staffs after every four calendar months. During the interviews all deaths, births, pregnancies and migrations that occurred since the previous visit are recorded, processed and stored in the database. The verbal autopsy (VA) method is used to assign cause of deaths that occurred within the study area [29]. VA interviews are conducted by trained field workers using standardized VA questionnaires. The main caregiver was interviewed about the signs and symptoms of the child’s terminal illness and care seeking behavior during the illness. During the study period, information from these forms was independently reviewed by a panel of at most three clinical officers to assign most probable cause of death [33].

### Socioeconomic status and insecticide treated net data

The socioeconomic indicators routinely collected in the HDSS (2002–2004) were used to generate socioeconomic index employing multiple correspondence analysis (MCA) on household assets. The analysis of socioeconomic assets has been described elsewhere in detail [34]. In brief, the household assets and characteristics included occupation of household head, primary source of drinking water, use of cooking fuel, ownership of in-house assets (lantern lamp, sofa, bicycle radio and television) and livestock possessions (poultry, pigs, donkey, cattle, sheep and goats). Household socioeconomic status (SES) index was calculated as a weighted average of the above assets and then grouped into five quintiles with the first quintile representing the poorest households followed by very poor, poor, less poor and the last household being least poor. The ITN data at household level were obtained from a one-time survey, carried within the study area in 2002 to access the ITN coverage.

### Entomological inoculation rate

The estimates of EIR used in this study have been described elsewhere in detail [35, 36]. In brief, *Anopheles* mosquitoes were collected monthly using Centers for Disease Control and Prevention light traps from 10 randomly selected houses each month from HDSS database along with four additional houses neighbouring each index house. In each house, a light trap was placed next to the sleeping place of an individual who was randomly chosen from the list of household members and mosquitoes were collected for two sequential nights. Captured female mosquitoes were then tested for the presence of circumsporozoite antigens using an enzyme linked immunosorbent assay method. Monthly high resolution estimates of EIR together with their prediction errors were obtained using Bayesian geostatistical zero inflated binomial and negative binomial predictive models [35, 36]. The models included environmental and climatic factors extracted from satellite data, harmonic seasonal trends and parameters describing space–time correlation.

### Analysis of EIR-mortality relationship

The analysis included all under-5 year old children who were residents between May 2002 and December 2004 as defined by HDSS residency rule [29]. These children were grouped into three categories namely neonates (0–28 days old), post neonates (29 days–11 months old) and child (1–4 years old). Time at risk for each child was defined as the number of months (days for neonate) that child was a resident during the study period and aged below 5 years old. Because period measures based on time at risk rather than cohort measures were used, the values of the infant mortality rates do not correspond to those that would be obtained by using the numbers of live-births as the denominator.

Exploratory analysis was carried out in STATA 10 (Stata Corporation US) to assess the bivariate relations of malaria transmission with both all-cause and malaria specific mortality. All covariates that were significant at 15% significant level were further included into a Bayesian geostatistical spatiotemporal conditional logistic regression model. Spatial correlation was modelled via village-specific random effects, which are considered as latent observations of a spatial Gaussian process. Correlations between any pairs of village locations were considered as an exponential function of their distance, irrespective of direction and modelled by the variance covariance matrix of the process [37]. Temporal correlation was modelled by introducing monthly random effects arising from an autoregressive Gaussian (AR) processes. Different orders were considered for the AR process ranging from between zero and four.

For estimating the relationship of mortality with EIR, time to death for each child was treated as discrete at monthly intervals. Cox proportional hazard models were fitted using binary logistic regression [38, 39] to estimate, for each month *t*, the probability *p*_*ijt*_ that child *i* at location *j* dies. The logistic model included a term in *x*_*jt*_, the estimated EIR for the corresponding location and time-interval. Different transformations of EIR such as logarithmic, categorization and fractional polynomial functions of different orders were assessed to account for non-linearity. The Akaike’s information criterion (AIC) [40] was used to select the best transformation of EIR, which was found to be logarithm of the EIR estimate for the month previous to the mortality outcome (incremented by 1, to allow inclusion of data for EIR = 0). The prediction error of the EIR estimate was introduced into the model as a measurement error in the covariate. Bayesian models were fitted in OpenBugs version 3.1.2 (Imperial College and Medical Research Council London, UK). A description of the Bayesian geostatistical formulation of this model is given in Additional file 1: A.

### Analysis of operating characteristics of verbal autopsies

The estimated malaria-mortality relations obtained from the exposure response relationship was used as the gold standard to calculate the attributable mortality separately for all-cause and for malaria-specific (by VA) deaths. For each recorded death, the excess risk attributable to malaria exposure (conditional on the set of covariates applicable at the time of death) was computed as:

$${\text{AR}}_{i} \,{ = }\,p_{ijt} - \tilde{p}_{ijt}$$

where and $\tilde{p}_{ijt}$ is the corresponding counterfactual value that *p*_*ijt*_ would have taken had the EIR been zero. AR_*i*_ is thus the difference between the model estimate of the probability of dying, at EIR *x*_*jt*_, and that at EIR 0. The computation of $\tilde{p}_{ijt}$ and AR_*i*_ is described in Additional file 1: B.

From the definition of conditional probability, the probability that the death of child *i* is malaria attributable, *a*_*i*_, was obtained by dividing AR_*i*_ (the probability that a malaria death occurred) by *p*_*ijt*_, the probability of any death, i.e.

$${\text{a}}_{i} \,{ = }\,\frac{{p_{ijt} - \tilde{p}_{ijt} }}{{p_{ijt} }}$$

The total number of excess deaths associated with exposure to malaria (that is, the malaria attributable mortality) was calculated as the sum, ∑*a*_*i*_, over all deaths. To provide the analysis of sensitivity, specificity and predictive values of the VA (Table 4), this step in the calculations was also carried out summing only over specific categories of deaths, i.e. those recorded as malaria in VAs, those in specific age groups, or in specific exposure categories. This provides estimates of the numbers of misclassified deaths in each category of VA outcome, without the need to diagnose each individual death as malaria or otherwise. Standard formulae for sensitivity, specificity and predictive values were then used to evaluate the performance of the VA.

## Results

During the study period, 32,709 children under 5 years of age children were included in the study, contributing 47,170 person-years at risk. There were 3793 deaths among these children (80.4 deaths per 1000 person years at risk). Verbal autopsies were available for 3107 of the deaths, with 670, 1234 and 1203 of the latter deaths occurring in 2002 (May–December only), 2003 and 2004, respectively. The median age at death was 11 months and 53% of the total deaths occurred in infants. Most of the time at risk was in the EIR category of 1–5 infectious bites per person per year, but there was substantial time at risk and also deaths at lower and higher EIRs than this (see Table 1 below).

**Table 1:** Distribution of time at risk and deaths by entomological inoculation rate

	Entomological inoculation rate (IBPPY)					Total
	EIR = 0	> 0– < 1	> 1–5	> 5– < 10	≥ 10	
Neonatal						
Malaria deaths VA	0	5	1	1	1	8
All_cause deaths	37	132	124	24	7	324
Person years at risk	147.1	429.8	421.8	78.8	14.7	1092.2
Post-neonatal						
Malaria deaths VA	39	107	131	21	16	314
All_cause deaths	185	493	505	100	54	1337
Person years at risk	649.0	3080.9	5099.3	561.9	130.0	9521.1
Child 1–4						
Malaria deaths VA	16	109	148	26	15	314
All_cause deaths	136	485	620	146	59	1446
Person years at risk	1393.4	8850.4	15816.3	1633.1	331.1	28024.3

**Fig. 1:**
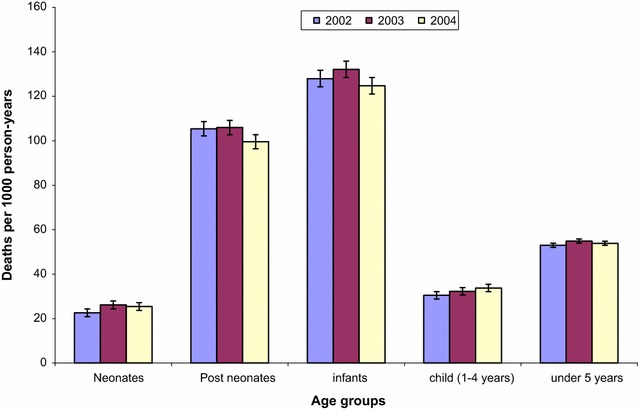
All-cause mortality by age groups. (Error bars are 95% confidence intervals)

Twenty percent of the deaths were reported in households in the poorest wealth quintile compared to 18% in least poor households. Implying that overall, the proportion of deaths did not vary by SES quintiles. Figure 1 shows all-cause mortality rates in each year of the study period. For instance, under-five mortality decreased from 55 (95% CI 54–56) deaths per 1000 person-years (pyrs) in 2003 to 54 (95% CI 53, 55) deaths per 1000 pyrs in 2004. Neonate and infant mortality rates also decreased. For example, infant mortality rate declined from 132 (95% CI 128, 136) to 125 (95% CI 121, 128) per 1000 person-years in the same period. However, child (1–4 years) mortality rate increased during the study period from 30 (95% CI 29, 32) deaths per 1000 pyrs in 2002 to 34 (95% CI 32, 35) deaths per 1000 person-years, in 2004. Similar trends were observed in each study region (Asembo and Gem).

**Fig. 2:**
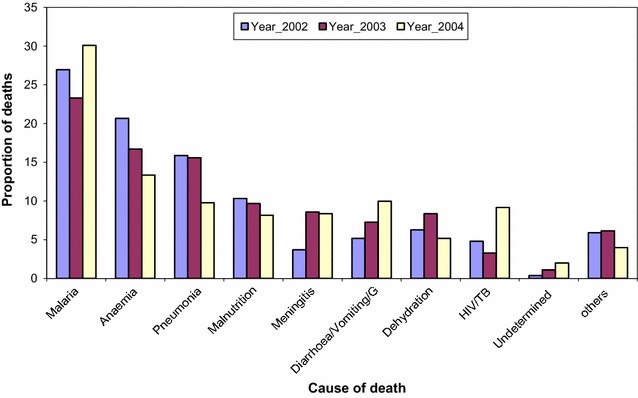
Main causes of death by VA among infants

**Fig. 3:**
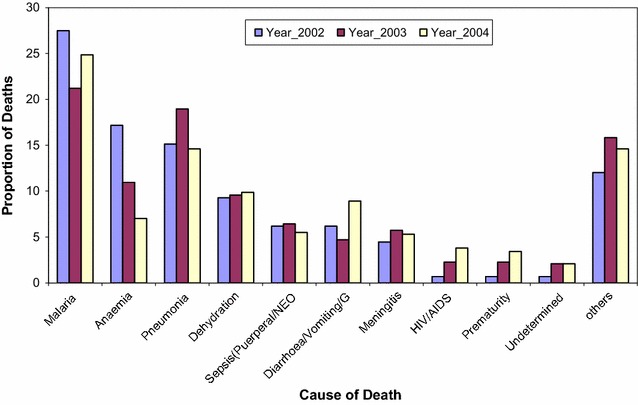
Main causes of death by VA among 1–4 year old children

Cause of death was assigned to 81% of the total deaths. The remainder (19%) is due to lack of respondent due to loss to follow up. Figures 2 and 3 depict the main causes of death for infants and child (1–4 years). Malaria was the main cause of death followed by anemia then pneumonia in the two age groups. A tendency for mortality to decrease over time was observed for all the main causes of death except for malaria, HIV and diarrhoea.

Table 2 presents the hazard ratio (HR) of predictors for all-cause and malaria specific mortality obtained from geostatistical, spatiotemporal models adjusted for EIR and SES. The EIR was strongly associated with all-cause mortality in all age groups and the relative risk of dying with any VA diagnosis was higher in children (1–4 years) (HR = 4.29, 95% CI 3.89, 4.73) compared to neonates (HR = 3.91, 95% CI 3.53, 4.32) and post-neonates (HR = 3.64, 95% CI 3.40, 3.89). Older children had lower all-cause mortality than infants, but there was no clear age trend after the first birthday.

**Table 2:** Hazard ratio (HR) estimates of predictors of all-cause and malaria specific mortality for under-five age categories from spatiotemporal models (models without ITN-use)

Covariate	All-cause mortality			Malaria specific mortality		
	Neonates HR (95% CI)	Post-neonate HR (95% CI)	Child (1–4 year HR (95% CI)	Neonates HR (95% CI)	Post-neonate HR (95% CI)	Child (1–4 year) HR (95% CI)
Constant	0.11 (0.07, 0.18)	0.02 (0.01, 0.04)	0.22 (0.10, 0.65)	0.10 (0.01, 0.40)	0.03 (0.01, 0.10)	0.04 (0.01, 0.09)
Age^a^	0.96 (0.94, 0.99)	0.94 (0.93, 0.95)	0.99 (0.98, 1.01)	1.01 (0.92, 1.10)	0.94 (0.91, 0.96)	0.99 (0.97, 1.02)
Log10 EIR	3.91 (3.53, 4.32)	3.64 (3.40, 3.89)	4.29 (3.89, 4.73)	3.97 (2.94, 4.89)	4.35 (3.72, 4.95)	4.29 (3.61, 5.06)
SES quintile						
1st	1.00	1.00	1.00	1.00	1.00	1.00
2nd	1.0 (0.89, 1.24)	0.94 (0.81, 1.08)	0.84 (0.62, 1.11)	1.00 (0.68, 1.49)	0.88 (0.66, 1.23)	0.75 (0.44, 1.34)
3rd	0.95 (0.82, 1.12)	0.88 (0.77, 1.02)	0.67 (0.50, 0.91)	1.01 (0.69, 1.51)	0.85 (0.62, 1.17)	0.55 (0.30, 1.00)
4th	0.65 (0.55, 0.78)	0.65 (0.56, 0.76)	0.65 (0.47, 0.87)	0.90 (0.60, 1.33)	0.74 (0.54, 1.02)	0.97 (0.56, 1.72)
5th	0.73 (0.62, 0.88)	0.71 (0.61, 0.83)	0.64 (0.49, 0.85)	0.98 (0.67, 1.48)	0.85 (0.62, 1.19)	0.71 (0.40, 1.28)
Random error	0.09 (0.06, 0.13)	0.10 (0.07, 0.14)	0.15 (0.09, 0.23)	0.23 (0.12, 0.41)	0.14 (0.08, 0.23)	0.22 (0.11, 0.45)
Spatial variation	0.15 (0.06, 0.34)	0.61 (0.18, 4.14)	0.23 (0.11, 0.53)	0.30 (0.13, 0.77)	0.22 (0.10, 0.57)	0.33 (0.14, 0.82)
Temporal variation	0.15 (0.08, 0.47)	0.13 (0.08, 0.24)	0.30 (0.13, 2.78)	0.63 (0.21, 7.14)	0.21 (0.12, 0.44)	0.22 (0.11, 0.48)
Spatial range (3/ρ)^b^	19.98 (1.11, 41.07)	26.64 (9.99, 41.07)	29.97 (6.88, 41.07)	15.54 (3.33, 39.96)	21.09 (3.33, 39.96)	19.98 (4.44, 39.96)

^a^Age is in month except for neonate group which is in day

^b^Minimum distance in kilometers at which spatial correlation is significant at 5%, *CI* credible intervals

Results from malaria specific mortality models (Table 2) showed that malaria exposure is associated with VA diagnosed malaria mortality in all age groups, with post-neonates (1–11 months) experiencing the highest relative risk (HR = 4.35, 95% CI 3.72, 4.95). Similarly age had a negative effect on VA diagnosed malaria mortality in post-neonates, but no trend was observed in neonates or children 1–4 years old. The estimated spatial correlation for both all-cause and malaria specific mortality was strong.

Comparison between the all-cause and malaria specific models shows that spatial range from the latter model had lower spatial ranges with narrower confidence intervals. Higher socioeconomic quintiles were associated with reduction in all-cause mortality in all age groups but no significant effects were observed in relation to malaria specific mortality.

The geostatistical spatiotemporal model that included ITN use data (Table 3) indicated that even after allowing for this important covariate, EIR was still associated with all-cause and malaria specific mortality, though the effect was not strong except in all-cause child (1–4 years) mortality (HR = 5.35, 95% CI 3.42, 7.86). Age was also associated with reduction in all-cause and malaria mortality in the first year of life (HR = 0.95, 95% CI 0.83, 0.99), but no trend was observed thereafter. ITN use was associated with reduction in all-cause and malaria-specific mortality in post-neonates and child (1–4 years) age groups. For instance, post-neonates who slept under an ITN were less likely to die due to any illness compared with their counterparts who did not use an ITN (HR = 0.15, 95% CI 0.02, 0.43). However, the protective effect of ITNs on mortality decreased with age (25 and 4% for all-cause mortality in post-neonates and children 1–4 years old, respectively).

**Table 3:** Posterior estimates of all-cause and malaria specific mortality for under-five age categories spatio-temporal models (models with ITN use)

Covariate	All- cause mortality		Malaria specific mortality	
	Post-neonates HR (95% CI)	Child (1–4 year HR (95% CI)	Post-neonates HR (95% CI)	Child (1–4 year HR (95% CI)
Constant	0.05 (0.01, 0.80)	0.04 (0.01, 0.5)	0.02 (0.00, 0.03)	0.05 (0.01, 0.37)
Age^a^	0.95 (0.83, 0.99)	1.01 (0.96, 1.04)	0.75 (0.41, 0.99)	0.96 (0.89, 1.03)
Log10 EIR	4.89 (2.42, 6.93)	5.35 (3.42, 7.86)	3.45 (1.36, 5.44)	4.54 (2.42, 7.56)
ITN use	0.75 (0.52, 0.97)	0.96 (0.76, 1.16)	0.80 (0.57, 1.10)	0.98 (0.64, 1.25)
Random error	0.56 (0.17, 2.70)	0.37 (0.16, 1.13)	0.62 (0.18, 1.66)	0.52 (0.17, 1.29)
Spatial variation	0.86 (0.18, 1.33)	0.52 (0.17, 1.04)	0.62 (0.18, 1.66)	0.60 (0.18, 1.29)
Temporal variation	0.72 (0.21, 1.48)	0.48 (0.18, 1.97)	0.65 (0.19, 1.40)	0.52 (0.18, 1.61)
Spatial range (3/ρ)^b^	11.10 (2.22, 34.41)	17.76 (3.33, 39.96)	26.64 (9.99, 41.07)	27.75 (9.99, 41.07)

^a^Age is in month except for neonate group which is in day

^b^Minimum distance in kilometers at which spatial correlation is significant at 5%, *CI* credible intervals

**Fig. 4:**
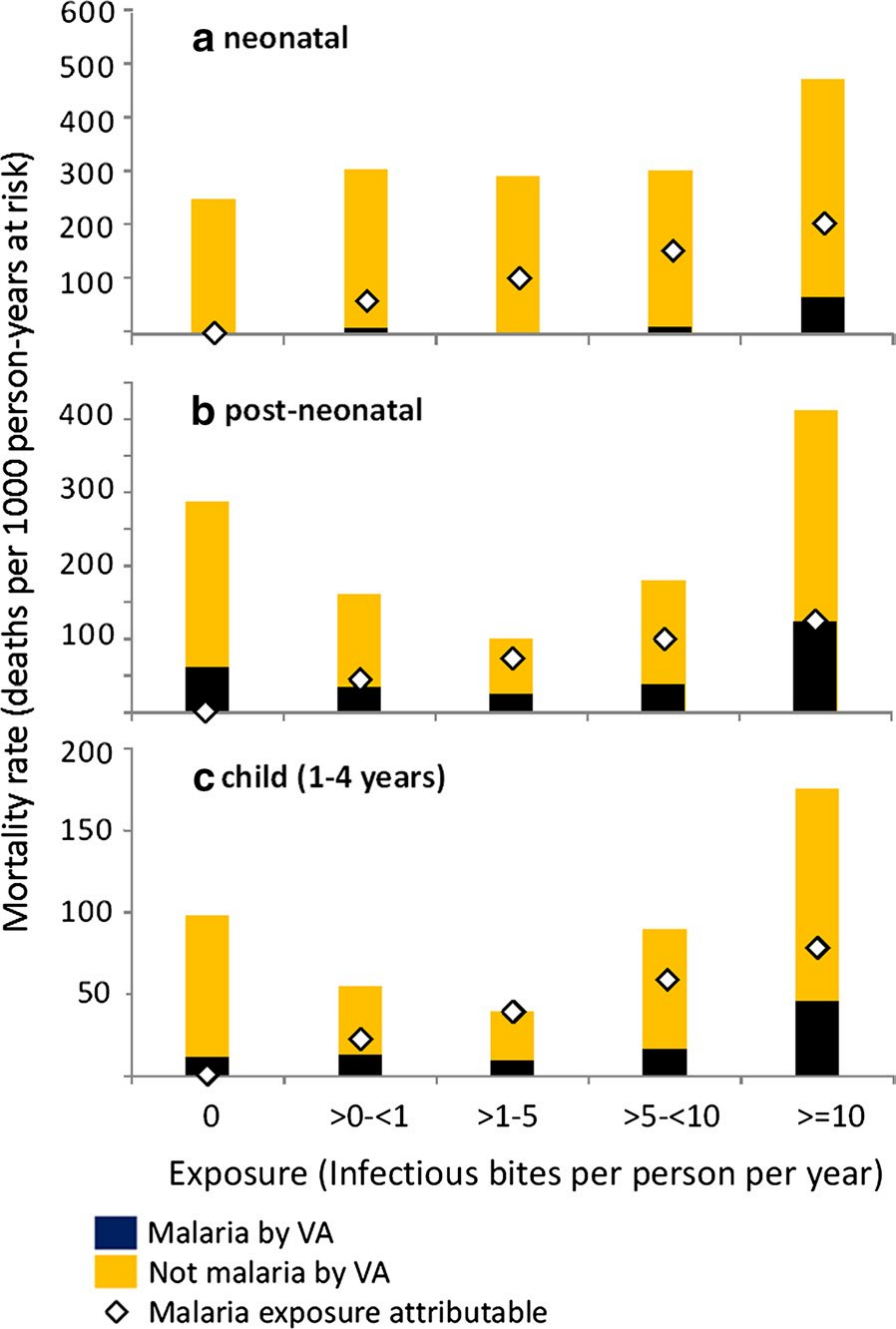
**a**–**c** Overall and excess mortality in relation to EIR for under-five age groups

Figure 4a, b and c depict the excess mortality (using the model without ITN) as a function of malaria exposure in relation to all-cause and malaria specific mortality for neonate, post-neonate and child age groups. Excess mortality is constrained to increase with malaria exposure in all age groups. The highest rate of excess all-cause and malaria specific mortality are reported in neonates and older children (1–4 years), respectively. Excess all-cause mortality rates are much higher than the overall rates of VA diagnosed malaria mortality.

In each age group, the total malaria attributable mortality estimated this way is substantially higher than the deaths assigned to malaria by VA so that while the 0–5 year rate of VA diagnosed malaria mortality was 16.5 deaths per 1000 pyrs of which most (10.2 deaths per 1000 pyrs) the total numbers of attributable deaths summed to 41.1 deaths per 1000 person years at risk were computed (Table 4).

**Table 4:** Diagnostic performance for malaria VA using the EIR-mortality relationship as gold standard

	Neo-natal	Post-neonatal	Child (1–4)	Overall
Estimates of numbers of deaths				
a: Malaria by VA, exposure attributable	7.7	141.1	243.1	391.9
b: Malaria by VA, not attributable	0.3	172.9	70.9	244.1
c: Other causes by VA, exposure attributable	64.3	436.3	605.0	1105.6
d: Other causes by VA, not attributable	251.7	586.7	527.0	1365.4
Derived quantities				
Overall all-cause mortality rate (deaths/1000 person-years) (from Table 1)	296.6	140.4	51.6	80.4
% of deaths assigned malaria as cause in VA (a + b)/(a + b + c + d) (%)	2.5	23.5	21.7	20.5
% of deaths attributable to malaria exposure (a + c)/(a + b + c + d) (%)	26.3	43.2	63.9	48.2
Malaria specific mortality rate estimated from VAs (deaths/1000 person-years)	7.3	33.0	11.2	16.5
Malaria exposure attributable mortality rate (deaths/1000 person-years)	78.0	60.7	33.0	38.8
Sensitivity of VA (a/(a + c))	0.11	0.24	0.29	0.26
Specificity of VA (d/(d + b))	1.00	0.77	0.88	0.85
Positive predictive value of VA (PPV) (a/(a + b))	0.96	0.45	0.77	0.62
Negative predictive value of VA (NPV) (d/(c + d))	0.80	0.57	0.47	0.55

For consistency across age groups, rates are expressed as deaths per 1000 person-years, rather than relative to numbers of live births (which is the usual convention for deaths in the first year of life)

Both analyses using VAs and exposure-attribution to assign cause of death indicate that malaria was much the most frequent cause of death among these children, but the analysis based on EIR attributes a far higher proportion of the mortality to malaria, with an overall malaria mortality rate estimated to be 38.8 deaths per 1000 person years at risk (Table 4). This is especially the case for neonatal deaths, of which only 2.5% were assigned to malaria by VA. Correspondingly, the sensitivity of the VA as compared with the model is low (averaging only 26%), and the estimated specificity surprisingly high (especially in the neonatal age group), with both positive and predictive values intermediate in value. The VA is thus very insensitive but with an upward trend in sensitivity with age (Table 4) while the estimated specificity of the VA is high, indicating that only a small number of deaths attributed to malaria in the VA would have occurred had malaria been absent.

## Discussion and conclusions

The present study assesses the effects of *P. falciparum* malaria exposure on both all-cause and evaluates the performance of VA in diagnosis malaria-attributed deaths in under-5 year old children in the KEMRI/CDC HDSS.

Both the bivariate and multivariate analyses indicate important positive association between all-cause and malaria specific mortality (by VA) with the malaria transmission intensity (EIR) of the previous month in each neonates, infants and children (aged 1–4 year). This implies that decreasing transmission intensity will reduce under-five all-cause and malaria specific mortality in the study area and particularly in infants, who experienced highest mortality rate (125 deaths per 1000 person-years). The positive association between malaria exposure and all-cause mortality is consistent with a large body of literature [23, 24], including a recent study [27] in a similar setting, that report the effect of malaria exposure decreases with age.

However, the large differences in the parameter estimates from those estimated from Rufiji [27] suggest that it would be premature to use the estimates from the present study for quantitative prediction of the effects of reducing transmission. There are several obvious potential confounders or effect modifiers, including SES, secular trends in unmeasured covariates such us HIV, and/or ITN use, that make it uncertain how generalizable are the estimates. Consistent with other studies in similar settings [41–43] higher SES quintiles were associated with lower all-cause mortality, and analyses that did not adjust for SES estimated higher apparent effects of malaria exposure (compare Tables 2 and 3). However SES was not an important determinant of malaria specific mortality rates by VA. Other sources of confounding cannot be excluded, though secular trends in unmeasured covariates also seem unlikely to have been a major confounder in the present study where the overall, decline in the under-five mortality during the study period was modest [14, 44]. There was a small age shift in the mortality with rates actually increasing over time in the older children (aged 1–4 years).

ITN use modifies the effect of EIR in a different way. The EIR estimates are calibrated against human landing collections, and are hence intended to provide unbiased estimates of the exposure of adults who are not using ITNs. The parameter for ITN use therefore measures the personal protection effect of the ITNs (based on only limited data from a single survey in 2002). In the post-neonatal age-group 100% ITN coverage was estimated to reduce all-cause mortality by 25%, with only a 3% reduction in all-cause mortality in the 1–4 year old age-range. Both the overall reduction [32, 45, 46] and age-dependence [27, 47] are of comparable magnitude to previously published estimates, including those from field trials. A previous study in the same area also reported that ITNs achieved a 22% reduction of all-cause mortality in post-neonates (1–11 months).

In agreement with other studies, both all-cause and malaria specific mortality increase less than proportionately with exposure, so that an increase from an EIR of 1 to 5 inoculations per month has a much larger effect than an increase from 6 to 10 inoculations. Malaria-specific mortality is expected to be more strongly associated with transmission intensity than all-cause mortality except in neonatal group, since the inclusion of deaths unrelated to malaria should bias the effect towards zero. However in this study the relative hazards were similar for both outcomes (Table 1). This is likely to be a consequence of the misclassification in the VA technique [19, 20].

Although it has been shown that physician-coded VA has low sensitivity and specificity in identifying malaria deaths in endemic areas, it remains the standard approach for ascertaining cause of death at community level in developing countries, where deaths mostly occur at home without any contact with the health system [19, 48, 49]. Recent computer-based expert algorithms and data driven (statistical) methods have been proposed as improvements in coding VAs but most of these methods are still under development and the enthusiasm for these methods currently exceeds that for physician based methods [50, 51]. The present study agrees with hospital validation exercises [22] in estimating a relatively high specificity of the VAs, suggesting that even if other pathogens were involved in the terminal illness, many of these children would not have died had it not been for a *P. falciparum* infection. The very low sensitivity of VAs in neonates suggests that most of the mortality attributed to malaria exposure in the youngest children, may well be secondary to maternal exposure (and hence indirect).

In general, it might be expected that VAs should over-report malaria deaths since malaria shares symptoms with other diseases including meningitis, typhoid, and acute respiratory-tract infections. Febrile illness with no other confirmed aetiology is generally recorded as malaria in VA [14, 19]. However the sensitivity estimates suggest that the VA captures only about one quarter of the deaths that would be averted by eliminating malaria, and that this proportion is even smaller in neonates. It has long been known that eliminating malaria reduces mortality rates by much more than the malaria diagnosable death-rate [52, 53], and these results are consistent with about half the malaria attributable deaths being indirect [23]. This would be consistent with the VA having a sensitivity of about one half in diagnosing direct malaria-specific mortality, as found in the hospital-based validations [22].

The very high estimates of almost 40 malaria attributable deaths per 1000 child years at risk, raises further fundamental issues about estimation of the burden of mortality. It suggests that interventions against malaria could potentially reduce overall child mortality by as much as twice the total direct malaria burden. The contribution of malaria interventions to recent massive improvements in child survival in East Africa has been unclear [54], partly because effects on this scale are much greater than those achieved in randomized controlled trials of ITNs [46]. If the results of the present study are generalizable across Africa, then it seems likely that malaria control could indeed have been responsible for most of the decline, largely as a result of reductions in indirect deaths.

## Additional file

**Additional file 1.** Statitstical details.

## References

[1] UNICEF (2009). The state of the world’s children.

[2] Bryce J, Boschi-Pinto C, Shibuya K, Black RE (2005). WHO estimates of the causes of death in children. Lancet.

[3] Black RE, Morris SS, Bryce J (2003). Where and why are 10 million children dying every year?. Lancet.

[4] Bhattarai A, Ali AS, Kachur SP, Mårtensson A, Abbas AK, Khatib R (2007). Impact of artemisinin-based combination therapy and insecticide-treated nets on malaria burden in Zanzibar. PLoS Med.

[5] Okiro EA, Hay SI, Gikandi PW, Sharif SK, Noor AM, Peshu N (2007). The decline in paediatric malaria admissions on the coast of Kenya. Malar J.

[6] Steketee RW, Sipilanyambe N, Chimumbwa J, Banda JJ, Mohamed A, Miller J (2008). National malaria control and scaling up for impact: the Zambia experience through 2006. Am J Trop Med Hyg.

[7] Bhatt S, Weiss DJ, Cameron E, Bisanzio D, Mappin B, Dalrymple U (2015). The effect of malaria control on *Plasmodium falciparum* in Africa between 2000 and 2015. Nature.

[8] Gething PW, Casey DC, Weiss DJ, Bisanzio D, Bhatt S, Cameron E (2016). Mapping *Plasmodium falciparum* mortality in Africa between 1990 and 2015. N Engl J Med.

[9] WHO (2009). World malaria report.

[10] Chambers RG (2012). UN Envoy’s response to estimates of global malaria mortality. Lancet.

[11] Murray CJL, Rosenfeld LC, Lim SS, Andrews KG, Foreman KJ, Haring D (2012). Global malaria mortality between 1980 and 2010: a systematic analysis. Lancet.

[12] Ye Y, Kyobutungi C, Ogutu B, Villegas L, Diallo D, Tinto H (2013). Malaria mortality estimates: need for agreeable approach. Trop Med Int Health.

[13] Colin DM, Doris MF, Mie I, Chalapati R, Alan DL (2005). Counting the dead and what they died from: an assessment of the global status of cause of death data. Bull World Health Organ.

[14] Abdullah S, Adazu K, Masanja H, Diallo D, Hodgson A, Ilboudo-Sanogo E (2007). Patterns of age-specific mortality in children in endemic areas of sub-Saharan Africa. Am J Trop Med Hyg.

[15] Greenwood BM, Greenwood AM, Bradley AK, Tulloch S, Hayes R, Oldfield FS (1987). Deaths in infancy and early childhood in a well-vaccinated, rural, West African population. Ann Trop Paediatr.

[16] Garenne M, Fontaine O (2006). Assessing probable causes of death using a standardized questionnaire: a study in rural Senegal. Bull World Health Organ.

[17] Murray CJL, Ortblad KF, Guinovart C, Lim SS, Wolock TM, Roberts DA (2014). Global, regional, and national incidence and mortality for HIV, tuberculosis, and malaria during 1990–2013: a systematic analysis for the Global Burden of Disease Study 2013. Lancet.

[18] Murray CJL, Vos T, Lozano R, Naghavi M, Flaxman AD, Michaud C (2012). Disability-adjusted life years (DALYs) for 291 diseases and injuries in 21 regions, 1990–2010: a systematic analysis for the Global Burden of Disease Study 2010. Lancet.

[19] Snow RW, Winstanley MT, Marsh VM, Newton C, Waruiru C, Mwangi I (1992). Childhood deaths in Africa: uses and limitations of verbal autopsies. Lancet.

[20] Todd JE, De Francisco A, O’Dempsey TJ, Greenwood BM (1994). The limitations of verbal autopsy in a malaria-endemic region. Ann Trop Paediatr.

[21] Quigley MA, Armstrong Schellenberg JR, Snow RW (1996). Algorithms for verbal autopsies: a validation study in Kenyan children. Bull World Health Organ.

[22] Korenromp EL, Williams BG, Gouws E, Dye C, Snow RW (2003). Measurement of trends in childhood malaria mortality in Africa: an assessment of progress toward targets based on verbal autopsy. Lancet Infect Dis.

[23] Ross A, Maire N, Molineaux L, Smith T (2006). An epidemiologic model of severe morbidity and mortality caused by *Plasmodium falciparum*. Am J Trop Med Hyg.

[24] Smith TA, Leuenberger R, Lengeler C (2001). Child mortality and malaria transmission intensity in Africa. Trends Parasitol.

[25] Snow RW, Marsh K (2002). The consequences of reducing transmission of *Plasmodium falciparum* in Africa. Adv Parasitol.

[26] Gemperli A, Vounatsou P, Kleinschmidt I, Bagayoko M, Lengeler C, Smith T (2004). Spatial patterns of infant mortality in Mali: the effect of malaria endemicity. Am J Epidemiol.

[27] Rumisha SF, Smith TA, Masanja H, Abdulla S, Vounatsou P (2014). Relationship between child survival and malaria transmission: an analysis of the malaria transmission intensity and mortality burden across Africa (MTIMBA) project data in Rufiji demographic surveillance system, Tanzania. Malar J.

[28] Krishna A, Kristjanson P, Radeny M, Nindo W (2004). Escaping poverty and becoming poor in 20 Kenyan villages. J Human Dev Capab.

[29] Odhiambo FO, Laserson KF, Sewe M, Hamel MJ, Feikin DR, Adazu K (2012). Profile: the KEMRI/CDC health and demographic surveillance system—Western Kenya. Int J Epidemiol.

[30] Bayoh MN, Mathias DK, Odiere MR, Mutuku FM, Kamau L, Gimnig JE (2010). *Anopheles gambiae*: historical population decline associated with regional distribution of insecticide-treated bed nets in western Nyanza Province, Kenya. Malar J.

[31] Gimnig JE, Kolczak MS, Hightower AW, Vulule JM, Schoute E, Kamau L (2003). Effect of permethrin-treated bed nets on the spatial distribution of malaria vectors in Western Kenya. Am J Trop Med Hyg.

[32] Lindblade KA, Eisele TP, Gimnig JE, Alaii JA, Odhiambo F, Ter kuile FO (2004). Sustainability of reductions in malaria transmission and infant mortality in western kenya with use of insecticide-treated bed nets: 4 to 6 years of follow-up. JAMA.

[33] Adazu K, Lindblade KA, Rosen DH, Odhiambo F, Ofware P, Kwach J (2005). Health and demographic surveillance in rural Western Kenya: a platform for evaluating interventions to reduce morbidity and mortality from infectious diseases. Am J Trop Med Hyg.

[34] Amek N, Vounatsou P, Obonyo B, Hamel M, Odhiambo F, Slutsker L, Laserson K (2015). Using health and demographic surveillance system (HDSS) data to analyze geographical distribution of socio-economic status; an experience from KEMRI/CDC HDSS. Acta Trop.

[35] Amek N, Bayoh N, Hamel M, Lindblade KA, Gimnig JE, Odhiambo F (2012). Spatial and temporal dynamics of malaria transmission in rural Western Kenya. Parasites Vectors.

[36] Amek N, Bayoh N, Hamel M, Lindblade KA, Gimnig J, Laserson KF (2011). Spatio-temporal modeling of sparse geostatistical malaria sporozoite rate data using a zero inflated binomial model. Spat Spatiotemporal Epidemiol.

[37] Diggle PJ, Moyeed RA, Tawn JA (1998). Model-based geostatistics. Appl Stat.

[38] Singer JD, Willett JB (1993). It’s about time: using discrete-time survival analysis to study duration and the timing of events. J Educ Behav Stat.

[39] Manda S, Meyer R (2005). Age at first marriage in Malawi: a Bayesian multilevel analysis using a discrete time-to-event model. J R Stat Soc Ser A.

[40] Akaike H (1974). A new look at the statistical model identification. IEEE Trans Autom Control.

[41] Po JYT, Subramanian SV (2011). Mortality burden and socioeconomic status in India. PLoS ONE.

[42] Sasiwongsaroj K (2010). Socioeconomic inequalities in child mortality: a comparison between Thai Buddhists and Thai Muslims. J Health Res.

[43] Wagstaff A (2000). Socioeconomic inequalities in child mortality: comparisons across nine developing countries. Bull World Health Organ.

[44] Hamel MJ, Adazu K, Obor D, Sewe M, Vulule J, Williamson JM (2011). A reversal in reductions of child mortality in western Kenya, 2003–2009. Am J Trop Med Hyg.

[45] Diallo DA, Cousens SN, Cuzin-Ouattara N, Nebié I, Ilboudo-Sanogo E, Esposito F (2004). Child mortality in a West African population protected with insecticide-treated curtains for a period of up to 6 years. Bull World Health Organ.

[46] Lengeler C (2004). Insecticide-treated bed nets and curtains for preventing malaria. Cochrane Database Syst Rev.

[47] Binka FN, Hodgson A, Adjuik M, Smith T (2002). Mortality in a seven-and-a-half-year follow-up of a trial of insecticide-treated mosquito nets in Ghana. Trans R Soc Trop Med Hyg.

[48] Van Eijk AM, Adazu K, Ofware P, Vulule J, Hamel M, Slutsker L (2008). Causes of deaths using verbal autopsy among adolescents and adults in rural western Kenya. Trop Med Int Health.

[49] Adjuik M, Smith T, Clark S, Todd J, Garrib A, Kinfu Y (2006). Cause-specific mortality rates in sub-Saharan Africa and Bangladesh. Bull World Health Organ.

[50] Reeves BC, Quigley M (1997). A review of data-derived methods for assigning causes of death from verbal autopsy data. Int J Epidemiol.

[51] Freeman JV, Christian P, Khatry SK, Adhikari RK, LeClerq SC, Katz J (2005). Evaluation of neonatal verbal autopsy using physician review versus algorithm-based cause-of-death assignment in rural Nepal. Paediatr Perinat Epidemiol.

[52] Giglioli G (1972). Changes in the pattern of mortality following the eradication of hyperendemic malaria from a highly susceptible community. Bull World Health Organ.

[53] Molineaux L, Lopez JV (1985). The impact of parasitic diseases and their control on mortality, with emphasis on malaria and Africa. Health policy, social policy and mortality prospects.

[54] O’Meara WP, Mangeni JN, Steketee R, Greenwood B (2010). Changes in the burden of malaria in sub-Saharan Africa. Lancet Infect Dis.

