# Supplementary material for: Infant and child mortality in relation to malaria transmission in KEMRI/CDC HDSS, Western Kenya: validation of verbal autopsy
Source: Malar J. 2018 Jan 18;17:37. doi: 10.1186/s12936-018-2184-x (PMC5774157; doi:10.1186/s12936-018-2184-x)
Supplement: Supplementary file 1 — Additional file 1. Statitstical details. [file 12936_2018_2184_MOESM1_ESM.docx]

**Additional file 1: Statistical details**

## 1: Geostatistical model

Let $Y_{ijt}$ be the mortality (all-cause or malaria specific) status of child $i$ at village$j$ and time interval $t$and $\underline{X}_{ij}$ be the covariates associated with child $i$ at location$j$. We assume that $Y_{ijt}$ arises from a Bernoulli distribution. That is $Y_{ijt}\sim\text{Be}(\text{p}_{ijt})$ where, $\text{p}_{ijt}$is the probability of the child dying during time interval $t$. We modelled spatial correlation via village-specific random effects (which is considered as latent observations of a spatial Gaussian process) on the logit, as:

(S1)

where $\theta_{j}$ measures the remaining non-spatial variation at village level, $\underline{\beta}$ is the vector of regression coefficients, with $\alpha$ the specific coefficient measuring the EIR effect. EIR, $x_{jt}$, has units of infectious bites per month, and is modelled on the log scale as a covariate with measurement errors. It is estimated by separate Bayesian geostatistical models, that is $\text{Log} \text{EIR}_{i}\sim N(\log\text{E}\widetilde{\text{I}}\text{R}_{i},\sigma_{ei}^{2})$ predicted at the household of child $i$where $\text{E}\widetilde{\text{I}}\text{R}$and $\sigma_{e}^{2}$correspond to the mean and variance respectively obtained from posterior prediction distribution of EIR at that household.

The mortality models assumed$\underline{\varphi}\sim\text{MVN}(0,\sum)$, where is the covariance matrix with elements accounting for the covariance between any pair of villages $k$ and $l$ irrespective of the direction (isotropy). Using an exponential correlation function, the covariance matrix is defined by $\sum_{kl}=\sigma_{1}^{2}\exp(-\rho d_{kl})$ where $\sigma_{1}^{2}$ is the spatial variation, $d_{kl}$ is the distance between villages $k$and $l$, and $\rho$ is the rate of correlation decay increasing distance. The minimum distance at which the spatial correlation is significant at 5% is called range and can be obtained from the value$3/\rho$ [55]. An exchangeable prior distribution for$\theta_{j}$, that is $\theta_{j}\sim N(0,\sigma_{2}^{2})$, was assumed.

Temporal correlation was modeled via the monthly random effects$\varepsilon_{t}$ (t=1……32) and assumed an autoregressive (AR) process. The deviance information criterion [56] was used to identify the best fitting order of the process, which was found to be one. Thus we considered that$\varepsilon_{t}\sim N(\gamma\varepsilon_{t-1},\sigma_{3}^{2})$ , and$\varepsilon_{1}\sim N(0,\frac{\sigma_{3}^{2}}{1-\gamma})$. The terms $\sigma_{3}^{2}$and $\gamma$ are the temporal variance and autocorrelation parameters respectively with $\gamma\in(-1,1).$

A Bayesian model formulation requires the specification of prior distributions for all model parameters. In particular, we choose a non-informative normal prior distribution with mean zero and large variance for the $\underline{\beta}$ parameters, regression coefficients$(\underline{\beta})$, an inverse gamma priors for $\sigma_{e}^{2} ,$ $\sigma_{1}^{2},$ $\sigma_{2}^{2}$ and$\sigma_{3}^{2}$. A gamma prior for$\rho$ and a uniform prior for $\gamma$, that is $\sigma_{e}^{2} ,$ $\sigma_{1}^{2},\sigma_{2}^{2},\sigma_{3}^{2},\sim IG(2.01,1.01)$, $\gamma\sim U(-1,1)$ and$\rho\sim G(0.1,0.1)$.

The model was fitted using Markov Chain Monte Carlo (MCMC) simulation algorithm in OpenBugs version 3.1.2 (Imperial College and Medical Council, London, UK) to estimate model parameters [57]. Starting with some initial values for the parameters, we run two chains sampler discarding the first 5000 iterations. Convergence was assessed by the Gelman-Rubin diagnostic [58].

## 2: Estimates of Malaria Attributable Risk

For each individual,$i$, with EIR $x_{jt}$, the probability of dying (from any cause) during time interval *t, $p_{ijt}$,*  with EIR is estimated using the logistic model (equation S1):

$\text{logit}(p_{ijt})=\alpha\text{log}(x_{jt}+1)+C_{ijt}$ (S2)

where .

Equivalently,$\frac{p_{ijt}}{1-p_{ijt}}=\exp\left(C_{ijt}\right)(x_{jt}+1)^{\alpha}$, and $p_{ijt}=\frac{1}{1+\exp\left(-C_{ijt}\right)(x_{jt}+1)^{\alpha}}$.

The corresponding excess probability of dying (the attributable risk, $\text{AR}_{i}$), was computed only for the time points and locations applicable if and when the individual died. This is the difference between the estimated probability of dying for EIR$x_{ijt},$ and the counterfactual probability, $\widetilde{p}_{ijt}$, that would have applied if the corresponding EIR was 0, i.e.

$\widetilde{p}_{ijt}=\frac{1}{1+\exp\left(-C_{ijt}\right)}$ (S3)

$\text{AR}_{i}\text{= }p_{ijt}-\widetilde{p}_{ijt}=\frac{1}{1+\exp\left(-C_{ijt}\right)(x_{jt}+1)^{-\alpha}}-\frac{1}{1+\exp\left(-C_{ijt}\right)}$ (S4)

# References

55. Ecker MD, Gelfand AE. Bayesian variogram modeling for an isotropic spatial process. Journal of Agricultural, Biological, and Environmental Statistics. 1997; 2:347–69.

56. Spiegelhalter DJ, Best NG, Carlin BP, Van Der Linde A. Bayesian measures of model complexity and fit. Journal of the Royal Statistical Society: Series B (Statistical Methodology). 2002; 64:583–639.

57. Gelfand AE, Ravishanker N, Ecker MD. Modeling and Inference for Point-Referenced Binary Spatial Data. In: Dey DK, Ghosh M and Ghosh S, editors. Generalized Linear Models: A Bayesian Perspective. Marcel Dekker Press 2000; 373-86

58. Gelman A, Rubin DB. Inference from Iterative Simulation Using Multiple Sequences. Statistical Science. 1992; 7:457–72.
